# AGS3 and Gα_i3_ Are Concomitantly Upregulated as Part of the Spindle Orientation Complex during Differentiation of Human Neural Progenitor Cells

**DOI:** 10.3390/molecules25215169

**Published:** 2020-11-06

**Authors:** Jackson L. K. Yip, Maggie M. K. Lee, Crystal C. Y. Leung, Man K. Tse, Annie S. T. Cheung, Yung H. Wong

**Affiliations:** 1 Division of Life Science and the Biotechnology Research Institute, Hong Kong University of Science and Technology, Clear Water Bay, Kowloon, Hong Kong 999077, China; lkyip@connect.ust.hk (J.L.K.Y.); mgleebiochem@yahoo.com.hk (M.M.K.L.); crystal.cyleung@gmail.com (C.C.Y.L.); mk.jack.tse@gmail.com (M.K.T.); anniecst@ust.hk (A.S.T.C.); 2 State Key Laboratory of Molecular Neuroscience, Hong Kong University of Science and Technology, Clear Water Bay, Kowloon, Hong Kong 999077, China

**Keywords:** AGS3, G protein, LGN, neurogenesis, NuMA, Ric-8A

## Abstract

Adult neurogenesis is modulated by many G_i_-coupled receptors but the precise mechanism remains elusive. A key step for maintaining the population of neural stem cells in the adult is asymmetric cell division (ACD), a process which entails the formation of two evolutionarily conserved protein complexes that establish the cell polarity and spindle orientation. Since ACD is extremely difficult to monitor in stratified tissues such as the vertebrate brain, we employed human neural progenitor cell lines to examine the regulation of the polarity and spindle orientation complexes during neuronal differentiation. Several components of the spindle orientation complex, but not those of the polarity complex, were upregulated upon differentiation of ENStem-A and ReNcell VM neural progenitor cells. Increased expression of nuclear mitotic apparatus (NuMA), Gα_i_ subunit, and activators of G protein signaling (AGS3 and LGN) coincided with the appearance of a neuronal marker (β-III tubulin) and the concomitant loss of neural progenitor cell markers (nestin and Sox-2). Co-immunoprecipitation assays demonstrated that both Gα_i3_ and NuMA were associated with AGS3 in differentiated ENStem-A cells. Interestingly, AGS3 appeared to preferentially interact with Gα_i3_ in ENStem-A cells, and this specificity for Gα_i3_ was recapitulated in co-immunoprecipitation experiments using HEK293 cells transiently overexpressing GST-tagged AGS3 and different Gα_i_ subunits. Moreover, the binding of Gα_i3_ to AGS3 was suppressed by GTPγS and pertussis toxin. Disruption of AGS3/Gα_i3_ interaction by pertussis toxin indicates that AGS3 may recognize the same site on the Gα subunit as G protein-coupled receptors. Regulatory mechanisms controlling the formation of spindle orientation complex may provide novel means to manipulate ACD which in turn may have an impact on neurogenesis.

## 1. Introduction

Neurogenesis is a highly regulated process by which new neurons are spawned from neural stem cells and progenitor cells, and it is absolutely essential for the development of the brain and our nervous system [1]. In adulthood, functional neurons can still be generated from specific brain regions such as the subgranular zone of the hippocampus. Manipulation of adult neurogenesis to generate new neurons may thus hold the key to curative therapy against neurodegenerative diseases. However, the molecular mechanism by which neural stem cells become mature neurons is not well understood. Because the population of neural stem cells is limited, it is critical for these specialized cells to maintain their own population while giving rise to new neurons. This is achieved by asymmetric cell division (ACD), a process through which the neural stem cell can achieve self-renewal in one of the two daughter cells, as well as differentiating into different corresponding progenitors and eventually into various neuronal types such as glial cells, oligodendrocytes and mature neurons [2,3]. Hence a thorough understanding of the initiation and regulation of ACD may offer the opportunity to manipulate neurogenesis for therapeutic purposes.

ACD is mainly regulated by two protein complexes that are evolutionarily conserved. They are the polarity complex which is made up of partition defective 6 (Par6), Par3 and atypical protein kinase C (aPKC) [4], and the spindle orientation complex composed of nuclear mitotic apparatus (NuMA), activator of G protein signaling (AGS) and Gα_i_ subunit [5]. The Par6/Par3/aPKC tripartite complex is conserved from worms to vertebrates and is responsible for establishing apical–basal polarity. Par3 is thought to provide anchorage to assemble the polarity complex at the apical–lateral border by binding Par6 and recruiting Par6-associated proteins including aPKC. In mammalian cells, spindle positioning is achieved by the binding of the spindle orientation complex to the polarity complex through the ankyrin protein inscuteable (mInsc) [6,7]. It is generally believed that the binding of Gα_i_ to an AGS protein (e.g., AGS3 and AGS5/LGN) increases the latter’s affinity for NuMA which, in turn, interacts with dynein and exert forces to orient the spindle [8,9,10]. These mechanistic insights were initially derived from invertebrate model systems (*Drosophila* and *C. elegans*) and they are difficult to validate in complex stratified vertebrate systems where the outcome of neural progenitor/stem cell division varies [3]. Moreover, the signals that trigger the assembly of the polarity and spindle orientation complexes and the fate of the individual components remain largely unknown. A thorough understanding of how these proteins are regulated is especially important in adult neurogenesis, because neural stem cells do not undergo ACD spontaneously. The involvement of Gα_i_ and AGS proteins in the spindle orientation complex adds another level of complexity to ACD since many G protein-coupled receptors (GPCRs) can modulate adult neurogenesis [11] while AGS proteins also participate in other neuronal functions such as drug addiction that involve G_i_-coupled receptors [12].

Since both polarity and spindle orientation complexes participate in ACD, monitoring their regulation during the differentiation of neural progenitor stem cells may provide clues to decipher the signals that initiate ACD. However, it remains highly challenging to quantitatively measure the functions of neural progenitor stem cells and their differentiated products. Nevertheless, a number of human neural stem cell lines have been developed as in vitro model systems for studying neurogenesis. Hence, we utilized two commercially available neural stem cell lines, ENStem-A cell [13] and ReNcell VM [14], to monitor the expression of ACD protein complexes during differentiation. ENStem-A cell is derived from a National Institutes of Health (NIH)-approved H9 human embryonic stem cell and it has been utilized in cell transplantation experiments on stroke-damaged rats [15]. ReNcell VM is derived from the ventral mesencephalon region of human fetal brain tissue. Different from ENStem-A^TM^ cell, this cell line is immortalized by retroviral transduction with the v-myc oncogene and it can be propagated for 45 passages before losing the ability to differentiate. ReNcell VM has been employed to study the effects of microRNA [16] as well as key signaling pathways [17,18] on neuronal differentiation. By utilizing these cell lines, we revealed an upregulation of components of the spindle orientation complex during neuronal differentiation and demonstrated the specificity of molecular interactions of G protein subunits within the complex.

## 2. Results

### 2.1. Expression Profile of ACD-Related Proteins during Differentiation

**Figure 1:**
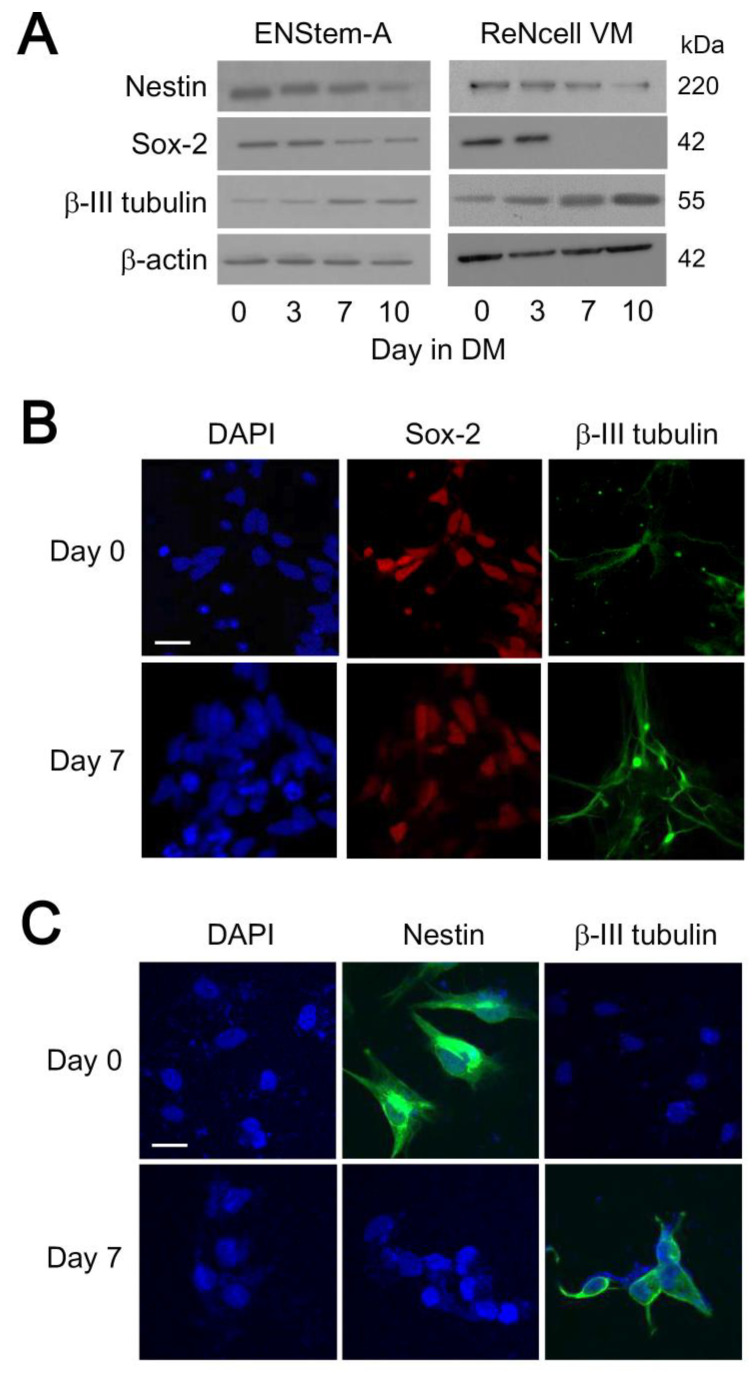
Differentiation of human neural progenitor cells. (**A**) ENStem-A cells were induced to undergo differentiation at passage number 6 while ReNcell VM cells were induced at passage number 9. Cell lysates were collected at Day 0, Day 3, Day 7 and Day 10 of differentiation and subjected to Western blot analysis using anti-Nestin, anti-Sox-2, anti-β-III tubulin and anti-β-actin antisera. Data shown represent one of three or more sets of immunoblots; other sets yielded similar results. (**B**) ENStem-A cells were fixed by 4% paraformaldehyde at Day 0 and Day 7 of differentiation. Cell nuclei were stained with DAPI (4’,6-diamidino-2-phenylindole) while Sox-2 and, β-III tubulin were identified by specific antisera and fluorescent secondary antibodies. Images were obtained with a Zeiss LSM700 confocal microscope. (**C**) ReNcell VM cells were processed as in B except anti-nestin was used instead of anti-Sox-2 for assessing pluripotency. Scale bar indicates 10 μm.

Prior to examining the expression profile of ACD-related proteins, we first characterized the differentiation capability of the two neural progenitor cell lines. Nestin and Sox-2 were utilized as markers for the pluripotency status while β-III tubulin served as a marker for differentiated neuronal cells. ENStem-A and ReNcell VM cells in early passage (around 6th to 9th passage after thawing) were induced to undergo differentiation for 10 days. Cells were harvested and lysed immediately upon initiation of differentiation (Day 0) or after culturing for 3, 7 or 10 days in the differentiation medium. The expressions of nestin and Sox-2 decreased over the course of differentiation in both cell lines (Figure 1A). The expression of Sox-2 was markedly reduced by Day 7, while the level of nestin became almost undetectable by Day 10. In contrast, the expression of β-III tubulin as a neuronal marker increased noticeably by Day 7 and beyond (Figure 1A). The down-regulation of nestin and Sox-2 with the concomitant appearance of β-III tubulin upon differentiation of the neural progenitor cells were further confirmed by confocal microscopy. As illustrated in Figure 1B, undifferentiated ENStem-A cells typically express Sox-2 but not β-III tubulin. Upon incubation of ENStem-A cells in the differentiation medium for 7 days, Sox-2 immunoreactivity was greatly reduced while those of β-III tubulin became clearly detectable (Figure 1B) , while the presence of glial fibrillary acidic protein was undetectable or negligible. Likewise, the expression of nestin in ReNcell VM became undetectable by Day 7 of differentiation, whereas β-III tubulin immunoreactivity appeared in the differentiated cells (Figure 1C). Under the conditions used, ReNcell VM would generate dopaminergic neurons and we indeed observed the appearance of tyrosine hydroxylase by Day 7 of differentiation (data not shown). These results indicate that a substantial proportion of the cell population underwent differentiation.

**Figure 2:**
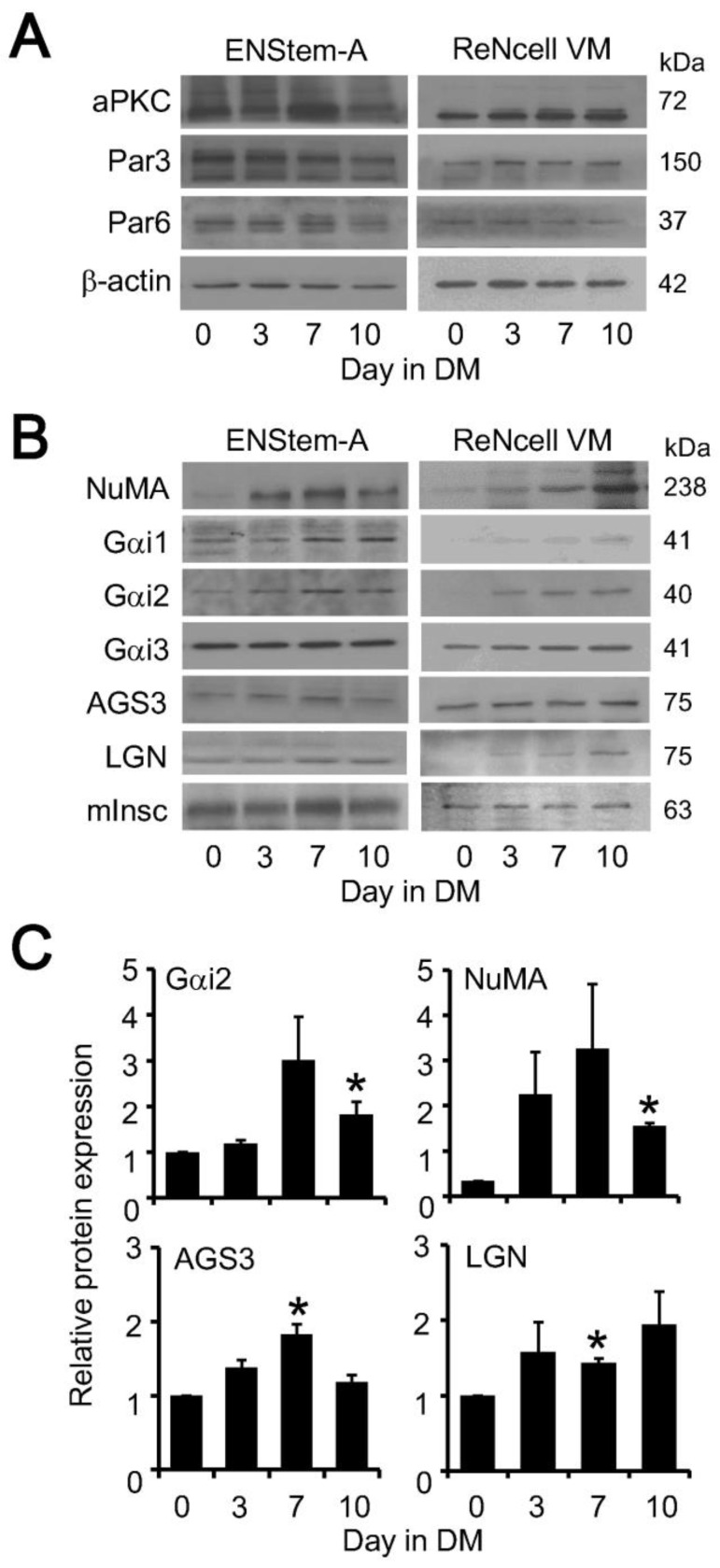
Upregulation of spindle orientation proteins in human neural progenitor cells during differentiation. ENStem-A and ReNcell VM cells were induced to undergo differentiation. Cell lysates were collected at Day 0, Day 3, Day 7 and Day 10 of differentiation. (**A**) Expression of polarity proteins were determined by Western blot analysis using anti-atypical protein kinase C (aPKC), anti-partition defective 3 (Par3), anti-Par6 and anti-β-actin antisera. (**B**) Expression of spindle orientation proteins were assessed by Western blot analysis using anti-nuclear mitotic apparatus (NuMA), anti-Gα_i1_, anti-Gα_i2_, anti-Gα_i3_, anti-AGS3, anti-LGN and anti-inscuteable antisera; please see β-actin immunoreactivity in panel A for reference protein expression. Data shown represent one of three or more sets of immunoblots; other sets yielded similar results. (**C**) Quantitative analysis of the expression of Gα_i2_, NuMA, AGS3 and LGN using the ImageJ software. Data were expressed as the mean ± S.E. of at least three independent sets of experiments. The probability of an observed difference being a coincidence was evaluated by Dunnett t test. Differences at values of *p* < 0.05 were considered significant (* *p* < 0.05).

Polarity proteins and spindle orientation proteins were then examined by Western blots during the differentiation of both neural progenitor cell lines. The expression of polarity proteins, namely aPKC, Par3, and Par6 in ENStem-A cells remained largely unchanged throughout the 10 days of differentiation (Figure 2A). Although variations in the levels of some target proteins were detected in independent experiments (e.g., elevated aPKC on Day 7 as shown in Figure 2A), such observations were not consistently reproduced. Likewise, no significant alterations in the levels of polarity proteins could be reproducibly detected in ReNcell VM cells over the 10-day differentiation period (Figure 2A). In contrast, several components of the spindle orientation protein complex were upregulated in both ENStem-A and ReNcell VM cells during the course of their differentiation (Figure 2B). NuMA was upregulated by Day 3 and it remained high throughout the differentiation of ENStem-A cells, while its upregulation appeared to be slower in ReNcell VM cells (Figure 2B). Even though it has been suggested that Gα_i3_ subunit is responsible for ACD [19], the identity of the Gα_i_ subunit involved in the spindle orientation complex has not been unequivocally established. Thus, we examined the expression of all three isoforms of the Gα_i_ protein by specific antisera. Gα_i1_ and Gα_i2_ were weakly expressed in ENStem-A cells but they were apparently up-regulated after 7 days of differentiation, whereas Gα_i3_ was more abundantly expressed with no change in its level throughout the differentiation (Figure 2B). Similar results were obtained with the ReNcell VM cells (Figure 2B). As the spindle orientation complex can be formed with either AGS3 or LGN, we monitored the expression of both proteins during the differentiation of the neural progenitor cell lines. AGS3 and LGN were both weakly expressed in ENStem-A cells and their levels were slightly elevated upon differentiation for 7 days or more (Figure 2B). Increased expressions of AGS3 and LGN were more discernable in ReNcell VM cells (Figure 2B). Expression of the ankyrin protein mInsc was not altered during the differentiation of ENStem-A or ReNcell VM cells (Figure 2B). Because some of the changes in target protein levels were rather subtle, immunoblots from multiple independent experiments were analyzed by densitometric scanning. As shown in Figure 2C, the levels of Gα_i2_, NuMA, AGS3, and LGN were significantly elevated in ENStem-A cells upon differentiation for 7 days or more.

**Figure 3:**
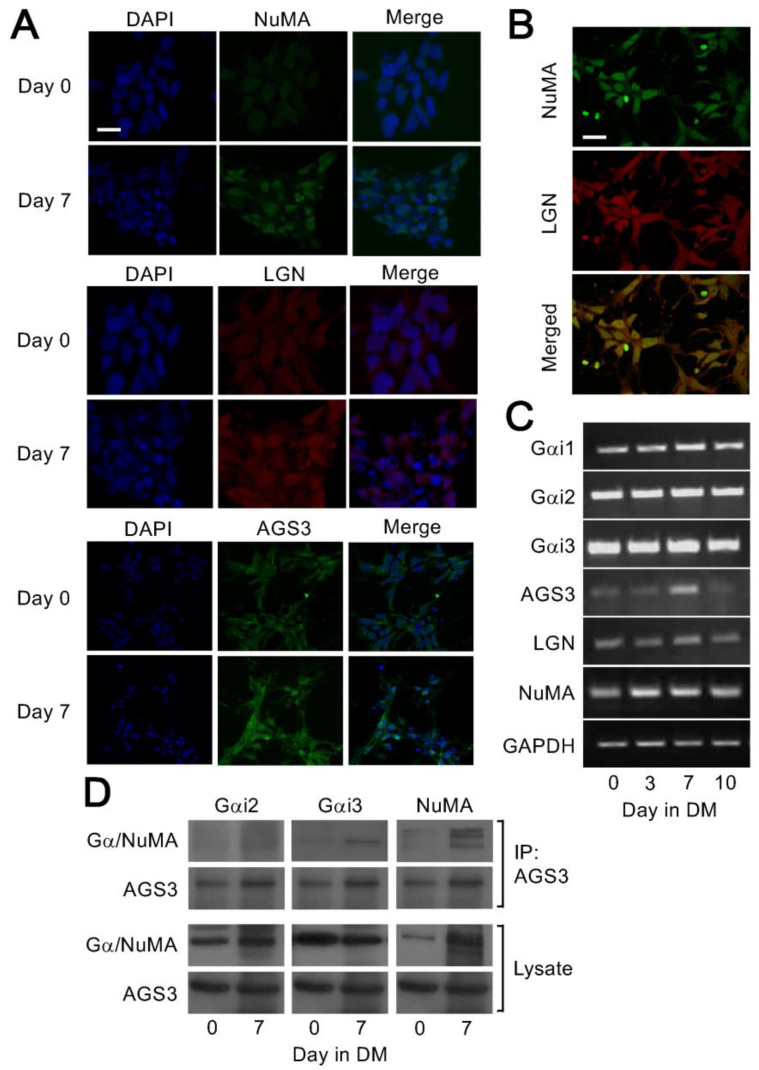
Co-localization and complex formation by upregulated spindle orientation proteins during differentiation of ENStem-A cells. (**A**) ENStem-A cells were fixed by 4% paraformaldehyde at Day 0 and Day 7 of differentiation. Cell nuclei were stained with DAPI and the presence of NuMA, LGN, and AGS3 detected by fluorescence staining using specific antisera. Images were obtained with a Zeiss LSM700 confocal microscope. Scale bar indicates 10 μm. (**B**) Images of LGN and NuMA at Day 7 were merged (bottom panel) to illustrate their co-localization. Scale bar indicates 10 μm. (**C**) ENStem-A cells were induced to undergo differentiation and total RNA was extracted using RNeasy Mini kit (Qiagen) at Day 0, Day 3, Day 7 and Day 10. Each RNA sample was then subjected to reverse transcription to generate cDNA followed by PCR amplification with primers corresponding to Gα_i1_, Gα_i2_, Gα_i3,_ AGS3, LGN, NuMA and GAPDH. (**D**) ENStem-A cells were induced to undergo differentiation and cell lysates were collected at Day 0 and Day 7 of differentiation. Samples were incubated with anti-AGS3 antisera and then immunoprecipitated using protein G agarose. Presence of Gα_i2_, Gα_i3_ and NuMA in the immunoprecipitates (IP) and cell lysates were detected by Western blotting with specific antisera.

We further used confocal imaging to examine how the expression and localization of these spindle orientation protein components changed during differentiation of ENStem-A cells. Indeed, more NuMA, LGN and AGS3 were detected at Day 7 as compared to Day 0 of differentiation, with LGN and AGS3 distributed throughout the cell and NuMA mainly found in the nucleus (Figure 3A). Co-localization of NuMA and LGN in cell nuclei was observed at Day 7 (Figure 3B). To investigate whether the protein expression changes were due to transcriptional or translational regulation, mRNA expression of each of the protein were checked by RT-PCR. The transcripts for all three Gα_i_ subunits and LGN remained essentially unchanged during differentiation, whereas the mRNA of AGS3 was transiently elevated at Day 7 and elevation of NuMA mRNA was detected from Day 3 onwards (Figure 3C). These results imply that the upregulation of spindle orientation proteins may be tightly correlated with the differentiation of neural progenitor cells.

To confirm that the spindle orientation proteins were indeed assembled as a complex during neuronal differentiation, co-immunoprecipitation experiment was performed in ENStem-A cells before and after differentiation for 7 days using AGS3 as the prey; AGS3 is preferred over LGN because it is restrictively expressed in neurons. Cell lysates with equivalent amount of AGS3 were subjected to immunoprecipitation with an anti-AGS3 antiserum and the precipitates probed with antisera against Gα_i2_, Gα_i3_, or NuMA. Although Gα_i2_ was clearly present in the cell lysates of Day 0 and Day 7 samples, it could not be detected in the AGS3 immunoprecipitates (Figure 3D). In contrast, Gα_i3_ was co-immunoprecipitated by anti-AGS3 after 7 days of differentiation but not on Day 0 (Figure 3D). Consistent with Figure 2B, NuMA was upregulated upon differentiation of ENStem-A cells and, like Gα_i3_, it was co-immunoprecipitated by anti-AGS3 (Figure 3D). These results indicate the formation of the AGS3/Gα_i3_/NuMA spindle orientation complex upon differentiation of neural progenitor cells.

### 2.2. Interaction between Gα_i_ Subunits and AGS3

**Figure 4:**
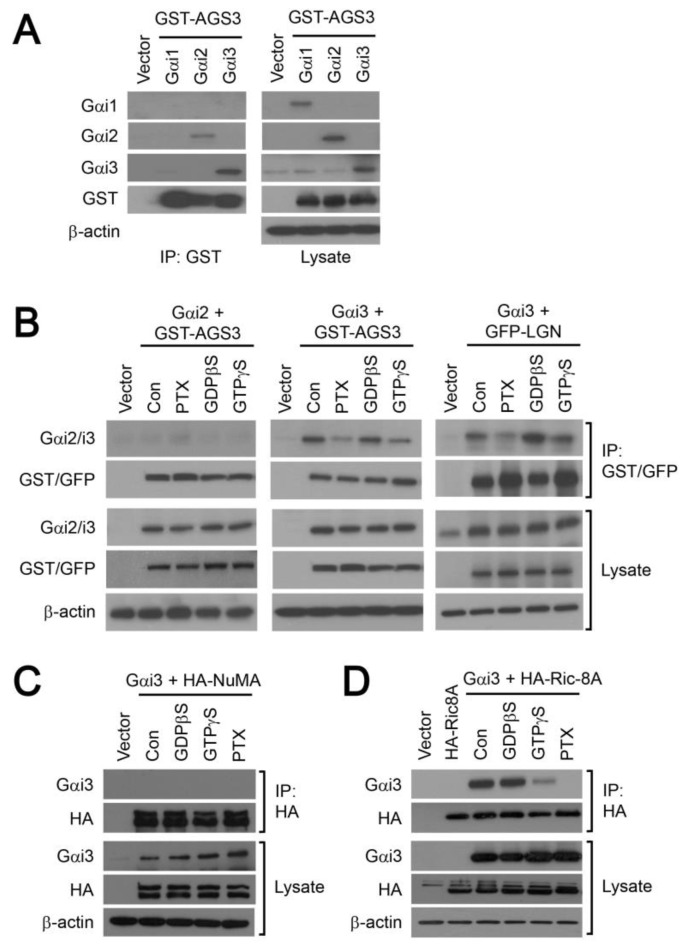
Interaction of Gα_i_ with components of the spindle orientation complex in HEK293 cells. (**A**) HEK293 cells were transiently transfected with vector (pcDNA3.1), or co-transfected with GST-tagged AGS3 and Gα_i1_, Gα_i2_ or Gα_i3_. Samples were incubated with anti-GST and then immunoprecipitated using protein G agarose. Presence of Gα_i1_, Gα_i2_, Gα_i3_ and GST-tagged AGS3 were analyzed by Western blotting with specific antisera. (**B**) HEK293 cells were transiently transfected with vector (pcDNA3.1), or co-transfected with GST-tagged AGS3 or GFP tagged LGN and Gα_i2_ or Gα_i3_. One day after transfection, cells was treated with or without pertussis toxin (PTX) for 16 h. Cell lysates were subjected to treatment with GDPβS and GTPγS (100 μM; 6 h at 4 °C). Samples were subjected to immunoprecipitation with either anti-GST or anti-GFP antisera and protein G agarose. Gα_i_ subunits in the immunoprecipitates were detected by Western blot analysis. (**C**) HEK293 cells transiently co-expressing HA-tagged NuMA and Gα_i3_ were subjected to co-immunoprecipitation with anti-HA agarose and Western blotting as in **B**. (**D**) HEK293 cells were treated as in ***C*** except that HA-tagged Ric-8A was transfected and probed instead of HA-tagged NuMA.

The preceding experiments revealed the upregulation of spindle orientation proteins during the differentiation of neural progenitor cells. The preferential interaction of AGS3 with Gα_i3_ is rather intriguing since Gα_i_ subunits share 85% sequence identity. Hence, we further examined the specificity of interactions among the spindle orientation proteins. To overcome the scarcity and difficulties in handling the neural progenitor cell lines, HEK293 cell was used as a model system to study protein–protein interactions by performing a series of co-immunoprecipitation experiments. HEK293 cells were transiently co-transfected with cDNAs encoding a GST-tagged AGS3 and one of the three Gα_i_ subunits, and the transfectants were subsequently subjected to immunoprecipitation with an anti-GST antibody. Among the three Gα_i_ subunits examined, both Gα_i2_ and Gα_i3_ were detected in the anti-GST immunoprecipitates with the latter exhibiting the strongest interaction with AGS3 (Figure 4A); Gα_i1_ failed to associate with AGS3 despite obvious overexpression of Gα_i1_ in the cell lysates of the transfectants (Figure 4A). These results suggest that AGS3 can distinguish between the different Gα_i_ subunits. Since AGS3/LGN are known to preferably bind to GDP-bound Gα_i_ subunits [8], the interaction between Gα_i3_ and AGS3 might be stabilized in the presence of the non-hydrolyzable GDPβS but weakened by GTPγS. In transfectants transiently co-expressing GST-AGS3 and Gα_i3_, treatment of the cell lysates with GTPγS (100 μM for 6 h at 4 °C) greatly reduced the amount of Gα_i3_ that was co-immunoprecipitated by the anti-GST antibody, whereas GDPβS treatment appeared to stabilize the formation of AGS3/Gα_i3_ as the level of Gα_i3_ found in the precipitates was identical to that of the control sample (Figure 4B, middle panels). Similar results were obtained with HEK293 cells co-expressing GFP-tagged LGN and Gα_i3_ (Figure 4B, right hand panels). In contrast, AGS3 failed to pull down Gα_i2_ from the cell lysates of transfectants co-expressing GST-AGS3 and Gα_i2_ (Figure 4B, left hand panels).

Recent structural studies have revealed that the Gα_i_ subunits bind to the four GoLoco motifs on AGS3/LGN through their switch I-III regions [20,21]. Since these regions are essential for the Gα_i_ to interact productively with GPCRs, Gβγ dimer, and effectors [22], their occupation by AGS3 is likely to displace other binding partners of Gα_i_. Conversely, the formation of AGS3/Gα_i_ complexes might be enhanced by limiting the ability of Gα_i_ to recognize its binding partners. GPCRs are the canonical and predominant partners of G_i_ proteins and their functional association can be abolished upon pertussis toxin (PTX)-catalyzed ADP-ribosylation of the Gα_i_ subunit. Hence, we examined the effect of PTX treatment (100 ng/mL, 16 h) on the ability of GST-AGS3 or GFP-LGN to pull down Gα_i3_ in HEK293 transfectants. Interactions between Gα_i3_ and both AGS3 and LGN were diminished following PTX treatment (Figure 4B, middle and right hand panels), indicating that the attachment of an ADP-ribose group on the C-terminus of Gα_i3_ disrupted the binding to AGS3/LGN. To further demonstrate the specificity of AGS3/Gα_i3_ interaction in an overexpression system, we asked if Gα_i3_ could be co-immunoprecipitated by NuMA and Ric-8A (a guanine nucleotide exchange factor or GEF of Gα_i_). In the spindle orientation complex, NuMA is bound to AGS3 but not Gα_i_ [5] and thus it is not expected to form a complex with Gα_i3_ in the absence of AGS3. Co-expression of HA-tagged NuMA with Gα_i3_ and subsequent immunoprecipitation with an anti-HA antibody failed to detect Gα_i3_ in the precipitates irrespective of pretreatments with guanine nucleotides of PTX (Figure 4C). Replacement of HA-NuMA by HA-Ric-8A in similar experiments resulted in the co-immunoprecipitation of Gα_i3_ with Ric-8A (Figure 4D). This is in line with a previous study which suggests that Ric-8A together with Gα_i_ are crucial in recruiting other components in the spindle orientation complex to the cell cortex for proper mitotic spindle orientation [10]. Like AGS3, the association of Ric-8A with Gα_i3_ was sensitive to GTPγS and PTX treatments (Figure 4D); failure of Ric-8A to form a complex with ADP-ribosylated Gα_i_ has been previously demonstrated [10]. These studies suggest that heterologous expression of Gα_i3_ with its binding partners such as AGS3 and Ric-8A can generate detectable complexes in HEK293 cells.

## 3. Discussion

Most of the mechanistic insights on ACD are derived from studies on invertebrate systems that allow in vivo monitoring of neurogenesis. Although imaging technology has advanced to a stage where it is now possible to broadly examine adult hippocampal neurogenesis in mice, it remains extremely challenging to follow the fate of specific cell lineages, let alone to determine the dynamic changes associated with the assembly and function of protein complexes. Our current understanding on ACD is largely based on studies in invertebrates such as *Drosophila* and *C.elegans* [6]. The existence of mammalian cell polarity and spindle orientation complexes have been extensively documented in studies utilizing recombinant proteins, and their putative functions demonstrated in several experimental systems such as epithelial cells that exhibit cell polarity [23,24,25]. Yet, the precise functions of these protein complexes in regulating ACD of neural progenitor cells are not fully understood. The use of two human neural progenitor cell lines in the present study enabled us to monitor the abundance of individual components that constitute the cell polarity and spindle orientation complexes. As the levels of Par3, Par6 and aPKC did not alter substantially during the 10-day differentiation period of both ReNcell VM and ENStem-A cells (Figure 2A), formation of the polarity complex with pre-existing components is apparently sufficient to support neurogenesis. In contrast, elevated expressions of all three components of the spindle orientation complex were detected towards the latter half of differentiation (Day 7 and Day 10) of the neural progenitor cells (Figure 2B), which coincided with the appearance of the neuronal marker β-III tubulin (Figure 1). Moreover, increased transcript levels of these components were observed in ENStem-A cells and the upregulated proteins were co-localized and capable of forming more complexes during differentiation (Figure 3). These results suggest that, unlike the polarity complex, pre-existing components may not be sufficient to ensure proper assembly of the spindle orientation complex during neurogenesis.

Neuronal differentiation and neurogenesis are intricately associated in order to generate new mature neurons. Instead of examining the effects of spindle orientation proteins on the process of ACD, our focus is placed on the assembly of the spindle orientation complex. Our data suggest that the formation of spindle orientation complex may be dynamically controlled during the differentiation of neural progenitor cells. Although all three components of the spindle orientation complex were upregulated upon differentiation of ReNcell VM and ENStem-A cells, elevation of NuMA was most easily detectable (Figure 2B). Co-immunoprecipitation data demonstrate that increased expression of NuMA during differentiation of ENStem-A cells can lead to the formation of additional complexes with AGS3 and Gα_i3_ (Figure 3D). The precise composition of the spindle orientation complex, however, remains equivocal as both AGS3 and LGN are capable of interacting with NuMA and various Gα_i_ subunits [25,26,27]. We used AGS3 primarily in the present study because its expression is restricted to neurons, whereas LGN is expressed in neuronal, astroglial, and microglial cultures [28]. It should be noted that an upregulation of AGS3 has been reported in differentiating human neural progenitor cells [29]. Although LGN appears to be involved in regulating spindle orientation during ACD [29], an alternative mechanism involving AGS3 regulating spindle positioning has also been proposed [26]. The mechanisms by which the components of the spindle orientation complex are upregulated have yet to be explored as both transcriptional and post-transcriptional processes may be involved. However, it should also be noted that increased expression of NuMA and AGS3 may not be exclusively associated with the establishment of spindle pole orientation because they possess additional functions; NuMA is apparently required for the selective induction of p53 genes [30] while the GoLoco (or G protein regulatory) motifs of AGS3 is critical for regulating cytokine production [31].

One of the unresolved issues regarding the spindle orientation complex in human neural progenitor cells pertains to the identity of the Gα_i_ subunit. Unlike *Drosophila* and *C. elegans* that only express one isoform of Gα_i_, mammalian genomes encode three different Gα_i_ subunits with 85–95% amino acid sequence identity. The Gα_i1-3_ share the same ability to inhibit adenylyl cyclase [32] and have partially overlapping expression patterns [33]. Gα_i2_ is expressed ubiquitously and represents the quantitatively predominant Gα_i_ isoform, but its expression is often accompanied by Gα_i1_ and/or Gα_i3_. All three subtypes of Gα_i_ were detected in both ReNcell VM and ENStem-A cells, albeit Gα_i3_ was more abundantly expressed. Although the levels of both Gα_i1_ and Gα_i2_ increased upon differentiation of the neural progenitor cells (Figure 2B), AGS3 appeared to preferentially bind Gα_i3_ in ENStem A cells (Figure 3D). The high abundance of Gα_i3_ might account for the lack of a detectable AGS3/Gα_i2_ complex in ENStem-A cells, because weak association of Gα_i2_ with AGS3 could be detected in transfected HEK293 cells (Figure 4A); the weaker interaction between AGS3 and Gα_i2_ further suggests a preference of Gα_i3_ by AGS3. A similar observation has also been reported in a study using mouse cerebral cortical progenitors, where AGS3 preferentially binds to Gα_i3_ instead of Gα_i1_ and Gα_i2_ [26]. Given the high sequence similarity and functional resemblance of the Gα_i_ subunits, it is difficult to comprehend why AGS3 prefers Gα_i3_. The inability of Gα_i1_ to interact with AGS3 in both ENStem-A (Figure 3D) and transfected HEK293 (Figure 4A) cells is especially intriguing, because it shares 95% amino acid sequence identity with Gα_i3_ [34]. Perhaps the answer lies in their differential availability within the intracellular compartments. It is noteworthy that, amongst the different Gα subunits, Gα_i3_ is the one most often detected in subcellular compartments in addition to its typical localization at the plasma membrane. Gα_i3_ is present on the cytoplasmic face of the Golgi cisternae [35], autophagosomes [36], and endocytic compartments [37], which may be associated to its non-canonical signaling functions in the control of cell differentiation [38,39] and autophagy [40]. Alternatively, the preference of AGS3 for Gα_i3_ may be dictated by other proteins that show selectivity for either or both protein partners. Unequivocal demonstration of the specificity of Gα_i3_ for AGS3 may require knockdown of the different Gα_i_ subunits. However, attempts to knockdown Gα_i_ subunits in neural progenitor cells resulted in non-viable cells (unpublished observations), while similar manipulations in HEK293 cells led to compensatory changes in the expression of G protein subunits [41].

**Figure 5:**
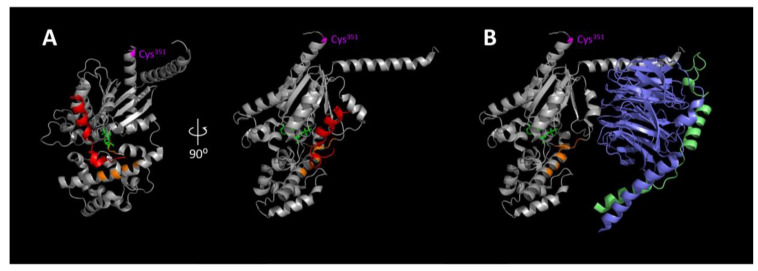
Crystal structures of Gα_i_-GDP in complex with GoLoco motif or Gβγ subunits. (**A**) Ribbon diagram of the Gα_i_-GDP in complex with GoLoco motif crystal structure in two views related by a 90° rotation about the vertical axis. The model of a full length Gα structure (residues 5-354) was constructed by combining published crystal structures of Gα_i_ subunits (PDB entries 4G5R and 1GP2). Selected amino acids suggested to participate in GoLoco motif interaction (Tyr^69^, Val^72^, Ser^75^, Gln^79^, Val^179^ and Thr^181^; Jia et al., 2012) are highlighted in *orange*. Gα_i_ and GoLoco peptide are shown in *light gray* and *red*, respectively. The PTX ADP-ribosylation site (Cys^351^) is highlighted in *magenta*. GDP is shown in the ball-and-stick model (*green*). (**B**) Ribbon diagram of the Gα_i_-GDP in complex with Gβγ subunits crystal structure. Gβ and Gγ subunits are shown in *blue* and *lime*. The color scheme of Gα_i_ is the same as in **A**.

Another interesting observation which deserves further discussion is the inability of ADP-ribosylated Gα_i_ to form complexes with AGS3, since PTX treatment is generally believed to have no effect on the function of AGS3 [42,43]. PTX targets the cysteine residue four amino acids from the C-terminus of Gα_i_ for ADP-ribosylation. The attachment of a bulky ADP-ribose group to the carboxyl α5 helix is known to prevent receptor recognition by the modified Gα_i_ subunit. Yet, as revealed by molecular modeling, the GoLoco motifs of AGS proteins bind to the switch II region of the Gα_i_ subunit [20,21] which is located on a plane different from the cysteine targeted by PTX (Figure 5A). The binding of the GoLoco motif on the Gα_i_ effectively prevents the latter from associating with Gβγ (Figure 5B) and thus provides a structural basis for the AGS proteins to primarily bind to Gβγ-dissociated Gα_i_ subunits [44]. The free Gα_i_ resulted from formation of the AGS3/Gα_i_ complex has been proven to be crucial in regulating spindle orientation as impairing Gβγ signaling and silencing AGS3 both disrupt the spindle orientation during ACD [26]. Yet, it is difficult to comprehend how ADP-ribosylation can impair the association of AGS3 to the Gα_i_ subunit. A plausible explanation may be related to the mechanism of ADP-ribosylation by PTX. Early studies have shown that PTX recognizes the heterotrimeric G_i_ proteins rather than the dissociated Gα_i_ subunit [45]. Since ADP-ribosylated G_i_ proteins cannot be stimulated by GPCRs, the generation of Gβγ-dissociated Gα_i_ subunits will diminish as the ADP-ribosylation reaction proceeds with time. With limited availability of free Gα_i_ subunits, formation of the AGS3/Gα_i_ complex will be impeded (Figure 4B). Inhibition of the binding of AGS3 to Gα_i_ by PTX was typically more extensive than that observed with GTPγS (Figure 4B). This may be attributed to mechanistic differences and distinct experimental conditions wherein the incubation time for GTPγS was substantially shorter (6 h versus 16 h). Alternatively, the C-terminal tail of Gα_i_ may represent another contact site for AGS3 because the current structural model of the AGS3/Gα_i_ complex is based on the GoLoco motif rather than the entire AGS3/LGN molecule [20,21]. This possibility cannot be discarded without resolving the complete structure of the AGS3/Gα_i_ complex. Since the PTX site on Gα_i_ is critical for receptor recognition, AGS3 binding to this site may result in competition with receptors for Gα_i_ subunits to modulate receptor signaling function. Such a scenario is highly likely in view of the recent demonstration of AGS3 forming a chemokine regulated, Gα_i_-dependent complex with CXCR4 and CCR7 receptors [46]. Indeed, there is substantial evidence in support of the notion that the Gα_i_-AGS3 complex is in close proximity to GPCRs and can regulate receptor signaling [47,48]. Possible involvement of GPCRs in regulating neurogenesis is further highlighted by the identification of numerous orphan GPCRs in neural stem cells including the ReNcell VM and ENStem-A cells [49].

Despite the general consensus that AGS3 selectively interacts with GDP-bound Gα_i_ subunits in the absence of the Gβγ dimers, the question of how GDP•Gα_i_ subunits are generated has not been unequivocally resolved. Activation of G_i_ proteins by a GEF will lead to the binding of GTP by Gα_i_ subunits and the concomitant release of Gβγ dimers. Intrinsic GTPase activity of the Gα_i_ subunit ensures the eventual hydrolysis of GTP to GDP and the resulting GDP•Gα_i_ subunits will then be available for binding by AGS3, provided that the latter can successfully compete against the Gβγ dimers. Such a mechanism is postulated to facilitate the formation of the spindle orientation complex, and the GEF is presumed to be Ric-8A because of its ability to associate with the spindle orientation complex [50]. However, the demonstration of Ric-8A acting as a GEF for G_i_ proteins was performed with recombinant proteins [50,51] and no such activity could be observed under in cellulo conditions [52]. It would seem that Ric-8A may not be the actual GEF that generates GDP•Gα_i_ subunits endogenously for the formation of the spindle orientation complex. Given that GPCRs can directly associate with AGS3 [47], they are well positioned to supply GDP•Gα_i_ subunits for AGS3. In this regard, it is noteworthy that several orphan GPCRs have significant effects on neurogenesis. GPR85, also known as the super conserved receptor expressed in brain 2 (SREB2), negatively regulates neurogenesis in the mouse hippocampus [53], whereas GPRC5B (G protein-coupled receptor class C 5B) appears to contribute to neurogenesis in the developing mouse neocortex [54], and Tre1 (Trapped in endoderm 1) orientates stem cell divisions through the spindle orientation and polarity complexes in the *Drosophila* [55]. In line with these recent studies supporting the possible involvement of GPCRs in neurogenesis, expression of GPR85 in ENStem-A cells is downregulated upon differentiation for 10 days (unpublished data). If orphan GPCRs are indeed involved in regulating the formation of spindle orientation complexes, their successful deorphanization may offer new avenues to manipulate neurogenesis.

While overexpressing different proteins involved in spindle orientation in HEK293 cell enables us to decipher the specific molecular organization of the protein complex, it is interesting to investigate the effect of disrupting interactions between heterotrimeric G protein and GPCRs/AGS3 on neurogenesis of human neural progenitors. One of potential future direction is to look at the spindle positioning when the human progenitor cell undergoes ACD. However, it is important to note that the use of PTX on neural progenitor cells may mask the investigation on structural interaction as it inhibits other GPCR-mediated pathways such as the phosphorylation of extracellular-signal regulated kinase that promotes cell proliferation and mitogen-activated protein kinase that leads to cell migration in human neural progenitor cells [56,57].

In summary, the present study revealed that the components of the spindle orientation complex are upregulated upon differentiation of human neural progenitor cells. Although several types of Gα_i_ subunits are endogenously expressed in human neural progenitor cells, Gα_i3_ appeared to be the preferred partner of AGS3 in the complex. Disruption of the AGS3/Gα_i3_ interaction by PTX further raised mechanistic implications in the formation of the spindle orientation complex. It is important to delineate the signals or conditions that direct GDP•Gα_i_ subunits to associate with AGS3 rather than GEFs that are abundantly expressed, and human neural progenitor cell lines may represent useful models for such endeavors.

## 4. Materials and Methods

### 4.1. Materials

The cDNAs of various human Gα subunits and AGS3 were purchased from the Missouri S&T cDNA Resource Center. HA-tagged mouse Ric-8A was kindly provided by Dr. Yijuang Chern (National Yang-Ming University). Cell culture reagents, including LipofectAMINE PLUS reagents were purchased from Invitrogen (Carlsbad, CA, USA). Anti-Sox-2 (AB5603), anti-β-III tubulin (MAB1637), anti-Par3 (07-330), anti-inscuteable (ABT64) and anti-nestin (MAB5326) antibodies were from Millipore (Darmstadt, Germany). Anti-β-actin (sab5500001) and anti-HA agarose (A2095) were obtained from Sigma-Aldrich (St. Louis, MO, USA). Antisera against Gα_i1_ (sc-13533), Gα_i2_ (sc-7276), Gα_i3_ (sc-262), aPKC (sc-216), AGS3 (sc-33222), Par6 (sc-25525) and GFP (sc-8334) were purchased from Santa Cruz Biotechnology (Santa Cruz, CA, USA). Anti-NuMA antibody (ab97585) was from Abcam (Cambridge, UK). Anti-GPSM2/LGN (PAB7195) was from Abnova (Taipei City, Taiwan). Anti-GST (#2625) antibody was purchased from Cell Signaling Technology (Danvers, MA, USA). GDPβS and GTPγS were from Calbiochem (San Diego, CA, USA). Protein G agarose was purchased from Roche Diagnostics (Indianapolis, IN, USA).

### 4.2. Cell Culture

ENStem-A and ReNcell VM were purchased from Millipore (Darmstadt, Germany). For culturing ENStem-A cell, plates were coated with 20 μg/mL poly-l-ornithine in sterilized water for 1 h and then coated with 5 μg/mL laminin in phosphate buffer solution (PBS) for another hour. Cells were maintained with ENStem-A expansion medium supplemented with 20 ng/mL fibroblast growth factor 2 (FGF-2) and 2 mM l-glutamate. For culturing ReNcell VM, plates were coated with 20 μg/mL laminin in Dulbecco’s Modified Eagle’s Medium with F12 for at least 4 h. They were maintained with ReNcell maintenance medium supplemented with 20 ng/mL of FGF-2 and epithelial growth factor 2 (EGF-2). HEK293 cells were obtained from the American Type Culture Collection (CRL-1573; Rockville, MD, USA) and maintained in Eagle’s minimum essential medium (HEK293) at 5% CO_2_, 37 °C with 10% fetal bovine serum, 50 units/mL penicillin and 50 μg/mL streptomycin.

### 4.3. Transfection of HEK293 Cell

HEK293 cells were seeded into 6-well plates at 5 × 10^5^ cells/well in culture medium the day before transfection. Various cDNAs at a concentration of 0.5 μg/well were transiently transfected into the cells using LipofectAMINE PLUS reagents following the supplier’s protocol. For co-immunoprecipitation assay, HEK293 cells were seeded into 100 mm culture plates and cultured to 60–80% confluence. Various cDNAs at a total concentration of 8 μg/plate were transiently transfected into the cells using LipofectAMINE PLUS reagents [58].

### 4.4. Differentiation of ENStem-A Cell and ReNcell VM

One day before the start of differentiation, cells were seeded at a density of 5 × 10^5^/mL in 60 mm plates. Day 0 represents the day when the ENStem-A expansion medium was replaced with ENStem-A differentiation medium without FGF-2 supplement, or when ReNcell VM maintenance medium was washed away with PBS and replaced with the maintenance medium without the addition of EGF-2 and FGF-2. On each time point where cell lysates were collected (i.e., Day 0, Day 3, Day 7 and Day 10), cells were lysed in lysis buffer (25 mM Hepes, 0.1% Nonidet *p*-40, 0.5% sodium deoxycholate, 1 mM dithiothreitol, 200 μM Na_3_VO_4_, 100 μM phenylmethylsufonyl fluoride, 2 μg/mL leupeptin, 4 μg/mL aprotinin, and 0.7 μg/mL pepstatin). Cell lysates were subjected to protein assay by Bio-Rad DC^TM^ protein assay kit in order to obtain samples with a similar protein amount for expression comparison.

### 4.5. Immunohistochemistry

Briefly, cells were first fixed with 4% paraformaldehyde, then permeabilized in 0.1–1% Triton X-100 in PBS (PBS-T), incubated in blocking buffer (5% bovine serum albumin), and incubated overnight at 4 °C with the following primary antibodies: mouse anti-β-III tubulin, mouse anti-Nestin, rabbit anti-AGS3, rabbit anti-NuMA, rabbit anti-Sox-2, or goat anti-LGN (1:1000). Species-specific Alexa Fluor secondary antibodies (1:1000; Invitrogen, Carlsbad, CA, USA) were used against the corresponding origin of primary antibodies. Cell culture coverslips were mounted on glass slides and stained with DAPI by ProLong Gold Antifade Mountant with DAPI (Invitrogen, Carlsbad, CA, USA). Images were captured by using confocal laser scanning microscope LSM700 (Zeiss, Oberkochen, Germany) as described previously [59].

### 4.6. Co-Immunoprecipitation Assays

Transfected HEK293 cells were washed with PBS twice. Cells were subsequently lysed in magnesium containing lysis buffer (25 mM HEPES, 50 mM MgCl_2_, 0.1% Nonidet *p*-40, 0.5% sodium deoxycholate, 1 mM dithiothreitol, 200 μM Na_3_VO_4_, 100 μM phenylmethylsufonyl fluoride, 2 μg/mL leupeptin, 4 μg/mL aprotinin, and 0.7 μg/mL pepstatin). Cell lysates were incubated with anti-HA affinity agarose gel or specific antisera followed by protein G agarose at 4 °C for 4 h. Immunoprecipitates were washed with 400 μL RIPA buffer 4 times and resuspended in 1× sample buffer. After boiling for 5 min, samples were subjected to the Western blot analysis.

### 4.7. Western Blot Analysis

Protein samples were resolved on 12% SDS-polyacrylamide gels and transferred to Osmonics nitrocellulose membrane. Resolved proteins were detected by their specific primary antibodies and horseradish peroxidase-conjugated secondary antisera. The immunoblots were visualized by chemiluminescence with the ECL kit from Amersham (Amersham, UK), and the images detected in X-ray films were quantified by densitometric scanning using the Eagle Eye II still video system (Stratagene, La Jolla, CA, USA). Western blots were quantified using ImageJ software (version 1.80).

### 4.8. RT-PCR

ENStem-A cells were washed twice with PBS followed by the addition of 500 μL TRIZOL reagent. Total RNA was extracted using the protocol supplied by the RNeasy Mini kit (Qiagen, Hilden, Germany). RNA (1 μg) of each sample was used for cDNA synthesis with random hexamer as primer by the SuperScriptTM III First-Strand Synthesis System (Invitrogen, Carlsbad, CA, USA). The cDNA product (1 μL) was then used in conjunction with 10 pmol of forward and reverse primers for PCR to determine the levels of target transcripts and GAPDH. The primer sequences targeting Gα_i1_, Gα_i2_, Gα_i3_, LGN, AGS3, NuMA and GAPDH are listed in Table 1. PCR (25 cycles each with 95 °C for 30 s, 55 °C for 60 s and 68 °C for 60 s) was carried out using KAPA 2G Fast HS Readymix PCR Kit (KAPA Biosystems, Wilmington, MA, USA). PCR products were then resolved using 1.2% agarose gel and the fragment sizes corresponding to Gα_i1_, Gα_i2_, Gα_i3_, LGN, AGS3, NuMA and GAPDH were 401 bp, 395bp, 355 bp, 498 bp, 308 bp, 581 bp and 206 bp, respectively.

### 4.9. Statistical Analysis

Data were expressed as the mean ± S.E. of at least three independent sets of experiments. The probability of an observed difference being a coincidence was evaluated by the Dunnett t test. Differences at values of *p* < 0.05 were considered significant.

## Figures and Tables

**Table 1:** Primer sequences used in detecting mRNA expression of corresponding genes by RT-PCR.

Gene	Sequence
Gα_i1_	Sense: 5′-GGA GTA AGA TGA TCG ACC GCA-3′ Antisence: 5′-AAG CTG GTA CTC TCG GGA TCT-3′
Gα_i2_	Sense: 5′-GAA GTT GCT GCT GTT GGG TG-3′ Antisnese: 5′-GAA GTT GCT GCT GTT GGG TG-3′
Gα_i3_	Sense: 5′-GAG CCA TGG GAC GGC TAA AG-3′ Antisnese: 5′-TGG CCA CCT ACA TCA AAC ATC T-3′
LGN	Sense: 5′-GCT GTC TGA CAT TGA CCT CCT-3′ Antisnese: 5′-ACC ACT AGC TTT CGC TTC CC-3′
AGS3	Sense: 5′-ATC TGA GCA TCG CCC AAG AG-3′ Antisnese: 5′-CTC CTT GTG GAA GGT CGT GG-3′
NuMA	Sense: 5′-GGA ACT GGC GAA GAT GAC CA-3′ Antisnese: 5′-AGA TGA CTG GCA AAC TCC CG-3′
GAPDH	Sense: 5′-GGC GTC TTC ACC ACC ATG GAG-3′ Antisnese: 5′-AAG TTG TCA TGG ATG ACC TTG GC-3′
